# The effect of introducing IGRA to screen French healthcare workers for tuberculosis and potential conclusions for the work organisation

**DOI:** 10.1186/1745-6673-8-12

**Published:** 2013-05-07

**Authors:** Adrien Moucaut, Albert Nienhaus, Benedicte Courtois, Virginie Nael, Claire Longuenesse, Bruno Ripault, Pierre Rucay, Stéphanie Moisan, Yves Roquelaure, Dominique Tripodi

**Affiliations:** 1 Department of Occupational Medicine and Occupational Hazards, University Hospital of Nantes, Nantes, France; 2 Laboratory of Ergonomics and Epidemiology in Occupational Health, LEEST-UA InVS - IFR 132- UPRES EA 4336, University of Angers, Angers, France; 3 Faculty of Medicine Medicine E, University Hospital, 4 rue Larrey, Angers cedex, F 49933, France; 4 Institute for Health Services Research in Dermatology and Nursing (IVDP), University Clinic Hamburg-Eppendorf, Hamburg, Germany; 5 Institution for Statutory Accident Insurance and Prevention in the Health and Welfare Services, Hamburg, Germany

**Keywords:** Tuberculosis, Healthcare workers, Interferon-gamma release assay

## Abstract

**Introduction:**

In France, pre-employment screening for tuberculosis (TB) is performed for healthcare workers (HCW). Screening is repeated when exposure to TB patients or infectious material occurs. The results of these TB screenings were analysed in a retrospective analysis.

**Method:**

Tuberculin skin tests (TST) and interferon-gamma release assays (QuantiFERON® Gold In-Tube – QFT) were used to perform the TB screenings. The screening results of 637 HCWs on whom QFT was performed were taken from the records of the University Hospital of Nantes.

**Results:**

In three (0.5%) HCW, the QFT was indeterminate. In 22.2%, the QFT was positive. A second QFT was performed in 118 HCWs. The reversion rate was 42% (5 out of 17). The conversion rate was 6% (6 out of 98). A TST was performed on 466 (73.5%) of the HCWs. Results for TST > 10 mm were 77.4%. In those with a TST < 10 mm, QFT was positive in 14% and in those with a TST ≥ 10 mm, QFT was positive in 26.7%. Depending on the definition for conversion in the QFT, the annual attack rate was 4.1% or 7.3%. X-ray and pneumology consultation was based on positive QFT rather than TST alone (52 out of 56). No active TB was detected.

**Conclusion:**

The TST overestimated the prevalence of LTBI in this cohort. The decision about X-ray and consultation regarding preventive treatment should be based on the QFT rather than the TST results. The high reversion rate should be taken into consideration when consulting with HCWs regarding preventive treatment. The high conversion rate seems to indicate that preventive measures such as wearing masks should be improved.

## Introduction

Healthcare workers’ (HCW) increased risk of contracting tuberculosis (TB) is well documented [1-3]. TB screening for HCWs is therefore considered a cornerstone for preventing TB in hospitals [4]. Until now, TB screening was performed using a tuberculin skin test (TST), which has several weaknesses, the most important being cross-reactivity with BCG vaccination, booster phenomena due to intradermal application and rather low sensitivity. The interferon-gamma release assays (IGRA) are a promising tool to overcome these problems [5-7]. Because IGRA use antigens specific to *Mycobacterium tuberculosis*, they do not show cross-reactivity with BCG vaccination and most non-tuberculose mycobacteria. As IGRA are in vitro tests, the problem of boosting in serial testing is circumvented. IGRA correlate better than TST with exposure to infectious patients and show a higher sensitivity for active TB than TST [8]. Furthermore, in low-incidence countries, IGRA have a higher predictive value for disease progression [9-11]. Therefore, they are likely to improve both the effectiveness and the efficiency of HCW screening [12]. However, interpretation of IGRA in the serial testing of HCWs remains to be clarified and a consensus needs to be found [7,13-17].

In France, the incidence of TB is low (8.9 cases per 100,000 in 2007), however the variability of the incidence rate is high. In the Paris region, the incidence rate is as high as 18.4/100,000. Therefore TB prevention is one of the highest public health priorities in France [18], and special focus is given to nosocomial infections and the screening of HCWs [19]. Since 2007, the French Health Authority has recommended the use of IGRA for these screenings. However, so far little is known about the results of these IGRA TB screenings for French HCWs. In the three small studies published so far, the rate of IGRA positivity was 12% to 32% [19-21], while a TST ≥ 10 mm was observed in 43% to 70% of HCWs. However, all three studies comprised less than 300 HCWs in total.

As recommended, IGRA were introduced at the University Medical Centre of Nantes, France, to screen HCWs for TB in 2007. In a retrospective study, TST and IGRA results were compared and the effects of the introduction of IGRA for TB screening on the number of X-rays, referrals to the pneumology department for further evaluation of TB and prescription of preventive treatments were analysed. The annual attack rate for latent TB infection (LTBI) was calculated based on different definitions of IGRA conversion.

## Method

The population of this cross-sectional study includes all workers at the University Hospital of Nantes, France, who participated in TB screening between May 2007 and May 2011 due to contact with infectious TB patients or materials or because of pre-employment screening and on whom an IGRA was performed. The University Hospital of Nantes is the largest hospital in the Nantes region and serves as a referral centre for TB patients throughout the region.

Results were assessed retrospectively from the subjects’ records. BCG vaccination for all newborns was mandatory in France and until 2008 was repeated if the TST was < 5 mm [22]. Therefore every HCW has been vaccinated at least once. All identified HCWs were French-born. As it was assumed that all HCWs were vaccinated, no data on BCG vaccination was considered in this analysis.

The TST was performed by trained personnel following standard procedures. In brief, 0.1 mL (2 TU) of purified protein derivate (Tubertest from SanofiPasteur) was injected intradermally at the volar side of the forearm and the transverse diameter of the induration was read after 72 to 96 hours. A diameter of ≥ 10 mm was considered positive. Following French guidelines [23], recent LTBI is considered to be very likely if the TST is ≥ 15 mm or the TST increase is ≥ 10 mm. Therefore these definitions for a positive TST were considered, too.

For the IGRA, the QuantiFERON® -TB Gold In-Tube assay (Cellestis Limited, Carnegie, Australia) (QFT) was administered in accordance with the manufacturer’s instructions. Data was extracted using a standardised data sheet. The IGRA was performed either as a confirmatory test for a positive TST or if a TST was contraindicated or refused. Retrospectively, it was not possible to assess why in particular an IGRA was performed and a TST was not performed. The ethics committee at the University Medical Centre of Nantes gave its consent to this anonymous retrospective data analysis.

### Statistical analysis

Chi-square tests were used for categorical data. Adjusted odds ratios (OR) and 95% confidence intervals (CI) were calculated for putative predictive variables using conditional logistic regression. Model building was performed backwards using the chance criteria for variable selection. For calculation of the annual attack rate, it was assumed that infections occurred continuously during the follow-up. Therefore the mean of the follow-up period was calculated in years and the annual attack rate is the cumulative conversion rate divided by this mean.

## Result

**Figure 1:**
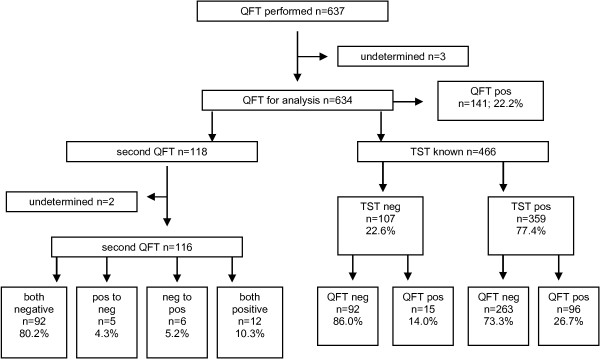
Flow chart of study population.

QFT results from 637 HCWs were assessed retrospectively. Three (0.5%) results were indeterminate, while positive QFT results accounted for 22.2% (Figure 1). The description of the study population with determinate QFT results is given in Table 1. Most HCWs were female (78.7%). Nurses constituted the largest single group (34.2). 12.8% of the HCWs worked in the pneumology department, 2.2% worked in the infectiology department and 21.3% in the emergency room.

**Table 1:** QFT results by demographic and occupational factors

	**QFT negative**		**QFT positive**		**All**		
	**N**	**%**	**n**	**%**	**N**	**%**	**p-value**
Female	386	77.4	113	22.6	499	78.7	
Male	107	79.3	28	20.7	135	21.3	0.637
Age (years)							
< 30	110	73.8	39	26.2	149	23.5	
30 – < 40	153	79.7	39	20.3	192	30.3	
40 – < 50	129	85.4	22	14.6	151	23.8	
50+	101	71.1	41	28.9	142	22.4	0.015
Status							
Temporary	59	67.0	29	33.0	88	13.9	
Student	45	86.5	7	13.5	52	8.2	
Staff	389	78.7	105	21.3	494	77.9	0.015
Profession							
Nurses	169	77.9	48	22.1	217	34.2	
Physicians	52	88.1	7	11.9	59	9.3	
Others	272	76.0	86	24.0	358	56.5	0.115
Department							
Pneumology	67	82.7	14	17.3	81	12.8	
Infectiology	10	71.4	4	28.6	14	2.2	
Emergency	105	77.8	30	22.2	135	21.3	
All others	311	77.0	93	23.0	404	63.7	0.656
TST							
< 10 mm	92	86.0	15	14.0	107	16.9	
10- < 15 mm	61	81.3	14	18.7	75	11.8	
+15 mm	202	71.1	82	28.9	284	44.8	
Unknown	138	82.1	30	17.9	168	26.5	0.003
TST increase							
No increase	44	67.7	21	32.3	65	10.3	
6- < 10 mm	17	65.4	9	34.6	26	4.1	
+10 mm	34	79.1	9	20.9	43	6.8	
Not tested	398	79.6	102	20.4	500	78.9	0.068
**Total**	**493**	**77.8**	**141**	**22.2**	**634**	**100.0**	

The TST was carried out on 466 HCW (73.5%). The TST result was ≥ 10 mm in 77.4% of the HCWs (Figure 1). In those with a TST < 10 mm, QFT was positive in 14.0% and in those with a TST ≥ 10 mm, QFT was positive in 26.7%. The probability of a positive QFT increased in line with the diameter of the TST. In those with a TST between 10 and < 15 mm, it was 18.7% and in those with a TST ≥ 15 mm it was 28.9% (Table 1). This relationship remained statistically significant after adjusting for age, gender, status, profession and department (Table 2). An increase in TST diameter between 6 and < 10 mm or ≥ 10 mm was not associated with an increased probability of a positive QFT (Table 1). No linear association between age and probability of positive QFT was observed.

**Table 2:** Adjusted odds ratios for a positive QFT depending on age und TST*

**Age (years)**	**OR**	**95%CI**
< 30	1	--
30 – < 40	0.7	0.4–1.3
40 – < 50	0.5	0.3–0.9
50+	1.3	0.8–2.2
TST		
< 10 mm	1	--
10- < 15 mm	1.4	0.6–3.1
+15 mm	**2.5**	**1.3–4.5**

*Status, TST increase did not meet the inclusion criteria.

The QFT was repeated in 118 HCWs. The median time between the two QFTs was 10.8 months (range: 0 to 47.5 months). Two QFTs were indeterminate (1.7%). 80.2% were negative in both QFTs and 10.3% were positive in both QFTs (Figure 1). The probability of conversion (negative to positive) and reversion (positive to negative) was dependent on the interferon-(INF)-gamma concentration in the first QFT (Table 3). Reversion occurred in 40% of those with INF-gamma between 0.35 and < 0.7 IU/mL and in 14% of those with a higher INF-gamma concentration in the first QFT. The total reversion rate was 29% and the conversion rate was 6%. Conversion occurred in 3.4% of those with a baseline INF-gamma concentration < 0.2 IU/mL and in 27% of those with a baseline concentration between 0.2 and < 0.35 IU/mL. If the definition of conversion is restricted to those with a baseline concentration below 0.2 IU/mL, and taking the time between the two tests into account, the annual attack rate for LTBI is 4.1%. In a simple negative to positive approach, the annual attack rate is 7.3% (no table).

**Table 3:** Results of second QFT depending on concentration of first QFT

	**Second QFT**					
	**Negative**		**Positive**		**All**	
**First QFT**	**N**	**%**	**n**	**%**	**N**	%*
< 0.2 IU/mL	85	97	3	3	88	76
0.2–0.35 IU/mL	8	73	3	27	11	10
**Negative QFT**	**93**	**94**	**6**	**6**	**99**	**86**
0.35- < 0.7 IU/mL	4	40	6	60	10	8
+0.7 IU/mL	1	14	6	86	7	6
**Positive QFT**	**5**	**29**	**12**	**71**	**17**	**14**

*Column per cent.

56 HCWs (52 [92.9%] with positive QFT) had a consultation with a pneumologist and 34 HCWs (32 [94.1%] with positive QFT) were prescribed preventive treatment. Four consultations and two preventive treatments were based on a positive TST even though the QFT was negative. No particular reason is known for this. Following a positive TST (≥ 15 mm or an increase ≥ 10 mm), 289 (out of 466, 62.0%) HCWs would have received a recommendation for a consultation with a pneumologist. Out of these 289 HCWs, 283 had a TST ≥ 15 mm and an additional six HCWs had a TST increase ≥ 10 mm but with a TST result < 15 mm (no table). X-rays performed by pneumologists did not reveal active TB. The median follow-up of the HCWs after the first QFT was 12 months (range: 0 to 48 months). No active TB was observed. Introducing QFT into the TB screening reduced recommendations for consultation with a pneumologist from 62.0% in the TST-tested subgroup to 8.8% in the whole group.

## Discussion

To our knowledge, this is the largest study comparing the results of TST and IGRA screening for HCWs in France. In HCWs considered to have a recent LTBI, following French recommendations for the interpretation of TST results [23], only 28.6% were positive according to the IGRA. No active TB was detected and no HCW with a positive TST or IGRA progressed to active TB. Therefore any decisions regarding further clinical evaluation and preventive treatment should be based on IGRA rather than TST alone.

A low confirmation rate of a positive TST was also reported in the three smaller French studies [19-21] and by other European HCW studies [24-26]. According to these, the probability of a positive IGRA increased in line with the diameter of the TST.

Prevalence of a positive IGRA was not associated with working in departments with a likelihood of contact with TB patients, e.g. pneumology, infectiology or emergency. Again, this agrees with the above-mentioned European studies which also failed to detect this association. In contrast to these prevalence studies, Rafiza and Rampal [27] observed an increased risk of LTBI for workers in the emergency department (RR 2.2; 95% CI 1.07–4.4) in their incidence study.

No linear trend between age and prevalence of positive IGRA was observed in our data. This is in contrast to data from Germany and Portugal [26,28], which describes a positive association between age and prevalence of positive IGRA. As our study population comprises HCWs on whom a QFT was performed, selection bias might be responsible for the failed detection of the expected association between age and LTBI prevalence. For the same reason, our data does not allow for an unbiased estimation of the LTBI prevalence in French HCWs. HCWs with a negative TST are not likely to be tested using QFT and therefore they are underrepresented in our study. However, as was shown in our data, probability of a positive IGRA is lower in those with a TST < 10 mm compared to those with a TST ≥ 10 mm.

Although only limited observations were available, conversion and reversion rates in our study were high and depended on concentrations in the first QFT. Again, this was reported by other authors, too [13,29-33]. Clinical decision-making should therefore consider the recent exposure situation and the concentration of the QFT. The reversion rate of QFT results between 0.35 und 0.7 IU/mL, albeit based on only a few observations, was high (40%). This corroborates the recommendation to use a borderline zone for the QFT of 0.2–0.7 IU/mL for interpretation of QFT in the serial testing of HCW [34]. Following this recommendation, a known, recent contact with an infectious TB patient and a concentration in the QFT (> 0.7 IU/mL instead of ≥ 0.35 IU/mL) make a recent infection likely.

Again based on just a few observations (6 out of 88), the annual attack rate for LTBI was 7.3% with a simple negative to positive interpretation of the IGRA, and 4.1% with a more prudent definition of a conversion (trespassing from < 0.2 IU/mL to > 0.7 IU/mL). This attack rate is about as high as the one reported for HCWs in Malaysia [27] and far higher than the attack rate observed in unexposed nursing students in Germany of < 1% [35], and it should be studied further. It indicates that protection of HCWs should be improved. As masks have proved to be an effective prevention measure, infectious patients, and HCWs in close contact with these patients, should be encouraged to wear masks [2].

## Conclusion

Introduction of IGRA in HCW screening will reduce the number of X-rays and the referrals to the pneumology department for further clinical evaluation. The annual attack rate for LTBI in our preliminary data gives rise to concerns. Preventive measures should therefore be improved, flows in early detection of infectious patients should be analysed, awareness of HCWs should be improved through training and masks should be worn more often, especially in emergency rooms as here patients are still undiagnosed.

## Consent

Written informed consent was obtained from the patient for publication of this report and any accompanying images.

## Abbreviations

BCG: Bacillus Calmette-Guérin; CI: Confidence interval; QFT: QuantiFERON gold in-tube; HCW: Healthcare worker; IGRA: Interferon-gamma release assay; INF: Interferon; LTBI: Latent tuberculosis infection; OR: Odds ratio; TB: Tuberculosis; TST: Tuberculin skin test.

## Competing interests

The authors declare that they do not have any conflicts of interest.

## Authors’ contributions

MA was engaged in data collection and gave considerable comments to the draft. NA performed the data analysis and drafted the paper. CB was engaged in data collection and gave considerable comments to the draft. NV was engaged in data collection and gave considerable comments to the draft. LC was engaged in data collection and gave considerable comments to the draft. RB was engaged in data collection and gave considerable comments to the draft. RP was engaged in data collection and gave considerable comments to the draft. MS was engaged in data collection and gave considerable comments to the draft. RY was engaged in data collection and gave considerable comments to the draft. TD conceived the study design, performed medical examinations and helped draft the paper. All the authors approved the final version of the paper.

## References

[B1] Baussano I Nunn P Williams B Pivetta E Bugiani M Scano F Tuberculosis among health care workers Emerg Infect Dis 2011 17 3 488 494 10.3201/eid1703.100947 21392441 PMC3298382

[B2] Menzies D Joshi R Pai M Risk of tuberculosis infection and disease associated with work in health care settings Int J Tuberc Lung Dis 2007 11 6 593 605 17519089

[B3] Seidler A Nienhaus A Diel R Review of epidemiological studies on the occupational risk of tuberculosis in low-incidence areas Respiration 2005 72 4 431 446 10.1159/000086261 16088290

[B4] Jensen PA Lambert LA Iademarco MF Ridzon R Guidelines for preventing the transmission of Mycobacterium tuberculosis in health-care settings, 2005 MMWR Recomm Rep 2005 54 1 141 16382216

[B5] Andersen P Doherty TM Pai M Weldingh K The prognosis of latent tuberculosis: can disease be predicted? Trends Mol Med 2007 13 175 182 10.1016/j.molmed.2007.03.004 17418641

[B6] Pai M Zwerling A Menzies D Systematic review: T-cell based assays for the diagnosis of latent tuberculosis infection: an update Ann Intern Med 2008 149 177 184 10.7326/0003-4819-149-3-200808050-00241 18593687 PMC2951987

[B7] Zwerling A van den Hof S Scholten J Cobelens F Menzies D Pai M Interferon-gamma release assays for tuberculosis screening of healthcare workers: a systematic review Thorax 2012 67 62 70 10.1136/thx.2010.143180 21228420

[B8] Diel R Goletti D Ferrara G Bothamley G Cirillo D Kampmann B Lange C Losi M Markova R Migliori GB Nienhaus A Ruhwald M Wagner D Zellweger JP Huitric E Sandgren A Manissero D Interferon-gamma release assays for the diagnosis of latent Mycobacterium tuberculosis infection: a systematic review and meta-analysis Eur Respir J 2011 37 1 88 99 10.1183/09031936.00115110 21030451

[B9] Torres Costa J Silva R Ringshausen F Nienhaus A Screening for tuberculosis and prediction of disease in Portuguese healthcare workers J Occup Med Toxicol 2011 6 1 19 10.1186/1745-6673-6-19 21658231 PMC3132202

[B10] Diel R Loddenkemper R Niemann S Meywald-Walter K Nienhaus A Negative and positive predictive value of a whole-blood interferon-γ release assays for developing active tuberculosis - An Update Am J Respir Crit Care Med 2011 183 88 95 10.1164/rccm.201006-0974OC 20802162

[B11] Diel R Loddenkemper R Nienhaus A Predictive value of interferon-gamma release assays and tuberculin skin testing for predicting progression from latent TB infection to disease state: a meta-analysis Chest 2012 142 1 1 13 10.1378/chest.12-1364 22490872

[B12] Nienhaus A Schablon A Costa JT Diel R Systematic review of cost and cost-effectiveness of different TB-screening strategies BMC Health Serv Res 2011 11 247 10.1186/1472-6963-11-247 21961888 PMC3196701

[B13] Ringshausen FC Schablon A Nienhaus A Interferon-gamma release assays for the tuberculosis serial testing of health care workers: a systematic review J Occup Med Toxicol 2012 7 6 10.1186/1745-6673-7-6 22537915 PMC3377540

[B14] Fong KS Tomford JW Teixeira L Fraser TG Vanduin D Yen-Lieberman B Gordon SM Miranda C Challenges of interferon-gamma release assay conversions in serial testing of health care workers in a tuberculosis control program Chest 2012 142 1 55 62 10.1378/chest.11-0992 22796839

[B15] Thanassi W Noda A Hernandez B Newell J Terpeluk P Marder D Yesavage JA Delineating a retesting zone using reciever operation characteristic analysis on serial QuantiFERON tuberculosis test results in US healthcare workers Pulm Med 2012 2012 291294 23326660 10.1155/2012/291294 PMC3544373

[B16] Nienhaus A Costa JT Screening for tuberculosis and the use of a borderline zone for the interpretation of the interferon-γ release assay (IGRA) in Portuguese healthcare workers J Occup Med Toxicol 2013 8 1 1 10.1186/1745-6673-8-1 23356875 PMC3563504

[B17] Nienhaus A Ringshausen FC Costa JT Schablon A Triopdi D IFN-γ release assay versus tuberculin skin test for monitoring TB infection in healthcare workers Expert Rev Ant Infect Ther 2013 11 1 37 48 10.1586/eri.12.150 23428101

[B18] Migueres B Carbonne A Abiteboul D Poirier C Bouvet E Astagneau P Tuberculosis among healthcare workers in northern France (2002–2007): descriptive analysis of notified cases and contact tracing Med Mal Infect 2010 40 524 529 10.1016/j.medmal.2010.02.007 20430555

[B19] Herrmann JL Simonney N Bergeron A Ducreux-Adolphe N Porcher R Rouveau M Allez M Leportier M Tazi A Lemann M Lagrange PH INFγ and antibody responses among French nurses during a tuberculosis contact tracing investigation Pathol Biol 2009 57 e49 e53 10.1016/j.patbio.2008.02.010 18395363

[B20] Faibis F Castelain D Moreau MC Tellier J Dekimeche A Ittah-Desmeulles H Fiacre A Demachy MC Prevalence of latent tuberculosis infection among health care workers from the emergency department of Meaux hospital using an interferon gamma release assay Presse Med 2011 40 e516 e520 10.1016/j.lpm.2011.03.014 21549552

[B21] Tripodi D Brunet-Court B Nael V Audrain M Chailleux E Germaud P Naudin F Muller JY Bourrut-Lacouture M Durand-Perdriel MH Gordeeff C Guillaumin G Houdebine M Raffi F Boutoille D Biron C Potel G Roedlich C Geraut C Schablon A Nienhaus A Evaluation of the tuberculin skin test and the interferon-gamma release assay for TB screening in French healthcare workers J Occup Med Toxicol 2009 4 30 10.1186/1745-6673-4-30 19948042 PMC2790451

[B22] Che D Lefebvre N Antoun F Fraisse P Depinoy M Antoine D Farge D Paty MC Tuberculosis in France: new challenges for the practitioners La Revue de Médecine Interne 2009 30 142 149 18845363 10.1016/j.revmed.2008.07.014

[B23] French guidelines for TB screening in HCWs Groupe de travail du conseil supérieur d’Hygiène publique: investigations à conduire autour d’un cas de tuberculose-maladie ou tuberculose-infection récente Revue des Maladies Infectieuses 2004 34 391 396 15622984

[B24] Nienhaus A Schablon A Le Bâcle C Siano B Diel R Evaluation of the interferon-gamma release assay in healthcare workers Int Arch Occup Enviro Health 2008 81 295 300 10.1007/s00420-007-0212-1 17605033

[B25] Ringshausen FC Schlosser S Nienhaus A Schablon A Schultze-Werninghaus G Rohde G In-hospital contact investigation among health care workers after exposure to smear-negative tuberculosis J Occup Med Toxicol 2009 4 1 11 10.1186/1745-6673-4-11 19505310 PMC2698921

[B26] Torres CJ Sa R Cardoso MJ Silva R Ferreira J Ribeiro C Miranda M Placido JL Nienhaus A Tuberculosis screening in Portuguese healthcare workers using the tuberculin skin test and the interferon-gamma release assay Eur Respir J 2009 34 6 1423 1428 10.1183/09031936.00053809 19948911

[B27] Rafiza S Rampal KG Serial testing of Malaysian health care workers with QuantiFERON®-TB Gold In-Tube Int J Tuberc Lung Dis 2012 16 2 163 168 10.5588/ijtld.11.0364 22236915

[B28] Schablon A Harling M Diel R Nienhaus A Risk of latent TB infection in individuals employed in the healthcare sector in Germany: a multicentre prevalence study BMC Infect Dis 2010 10 107 10.1186/1471-2334-10-107 20429957 PMC2877045

[B29] Pai M Joshi R Dogra S Mendiratta DK Narang P Kalantri S Reingold AL Colford JM Jr Riley LW Menzies D Serial testing of health care workers for tuberculosis using interferon-gamma assay Am J Respir Crit Care Med 2006 174 349 355 10.1164/rccm.200604-472OC 16690977 PMC2648116

[B30] Pai M Elwood K Interferon-gamma release assays for screening of health care workers in low tuberculosis incidence settings: dynamic patterns and interpretational challenges Can Respir J 2012 19 81 83 22536575 10.1155/2012/420392 PMC3373289

[B31] Ringshausen FC Nienhaus A Torres CJ Knoop H Schlosser S Schultze-Werninghaus G Rohde G Within-subject variability of mycobacterium-tuberculosis-specific interferon-gamma responses in German health care workers Clin Vaccine Immunol 2011 18 7 1176 1182 10.1128/CVI.05058-11 21593237 PMC3147310

[B32] Schablon A Harling M Diel R Ringshausen FC Torres Costa J Nienhaus A Serial testing with an interferon-gamma release assay in German healthcare workers GMS Krankenhhyg Interdiszip 2010 5 2 1 8 10.3205/dgkh000148 PMC2951105 20941341

[B33] Torres Costa J Silva R Sa R Cardoso MJ Nienhaus A Serial testing with the interferon-gamma release assay in Portuguese healthcare workers Int Arch Occup Enviro Health 2011 84 4 461 469 10.1007/s00420-010-0571-x PMC3058548 20721576

[B34] Nienhaus A Ringshausen FC Torres Costa J Schablon A Tripodi D INF-γ release assay versus tuberculin skin test for monitoring TB infection in healthcare workers Expert Rev Anti Infect Ther 2013 11 1 37 48 10.1586/eri.12.150 23428101

[B35] Schablon A Diel R Diner G Anske U Pankow W Ringshausen FC Nienhaus A Specificity of a whole blood IGRA in German nursing students BMC Infect Dis 2011 11 245 10.1186/1471-2334-11-245 21929799 PMC3189894

